# Thrombocytopenia After Transcatheter Valve-in-Valve Implantation: Prognostic Marker or Mere Finding?

**DOI:** 10.21470/1678-9741-2018-0078

**Published:** 2018

**Authors:** Renato C. de Souza, Leonardo Paim, Guilherme Viotto, Joaquim Aprigio, Lucas L. Araújo, Henrique Ribeiro, Roney O. Sampaio, Flavio Tarasoutchi, Pablo M. A. Pomerantzeff, José Honório Palma, Fabio B. Jatene

**Affiliations:** 1 Cardiovascular Surgery Division, Instituto do Coração do Hospital das Clínicas da Faculdade de Medicina da Universidade de São Paulo (InCor-HCFMUSP), São Paulo, SP, Brazil.

**Keywords:** Thrombocytopenia, Transcatheter Aortic Valve Replacement, Heart Valve Prosthesis Implantation, Heart Valves/Surgery

## Abstract

**Objective:**

To analyze the behavior of platelets after transcatheter valve-in-valve implantation for the treatment of degenerated bioprosthesis and how they correlate with adverse events upon follow-up.

**Methods:**

Retrospective analysis of 28 patients who received a valve-in-valve implant, 5 in aortic, 18 in mitral and 5 in tricuspid positions. Data were compared with 74 patients submitted to conventional redo valvular replacements during the same period, and both groups' platelet curves were analyzed. Statistical analysis was conducted using the IBM SPSS Statistics(r) 20 for Windows.

**Results:**

All patients in the valve-in-valve group developed thrombocytopenia, 25% presenting mild (<150.000/µL), 54% moderate (<100.000/µL) and 21% severe (<50.000/µL) thrombocytopenia. The platelet nadir was on the 4^th^ postoperative day for aortic ViV, 2^nd^ for mitral and 3^rd^ for tricuspid patients, with the majority of patients recovering regular platelet count. However, the aortic subgroup comparison between valve-in-valve and conventional surgery showed a statistically significant difference from the 7^th^ day onwards, where valve-in-valve patients had more severe and longer lasting thrombocytopenia. This, however, did not translate into a higher postoperative risk. In our study population, postoperative thrombocytopenia did not correlate with greater occurrence of adverse outcomes and only normal preoperative platelet count could significantly predict a postoperative drop >50%.

**Conclusion:**

Although thrombocytopenia is an extremely common finding after valve-in-valve procedures, the degree of platelet count drop did not correlate with greater incidence of postoperative adverse outcomes in our study population.

**Table:** 

Abbreviations, acronyms & symbols				
AKI	= Acute kidney injury		MODS	= Multiple Organ Dysfunction Score
BAV	= Balloon aortic valvuloplasties		PCI	= Percutaneous coronary interventions
CABG	= Coronary artery bypass grafting		SAVR	= Surgical aortic valve replacement
CPB	= Cardiopulmonary bypass		SOFA	= Sequential organ failure assessment
HIT	= Heparin-induced thrombocytopenia		TAVR	= Transcatheter aortic valve replacement
IABP	= Intra-aortic balloon pumps		VARC-2	= Valve Academic Research Consortium
ICU	= Intensive care unit		ViV	= Valve-in-valve
LODS	= Logistic Organ Dysfunction Score			

## INTRODUCTION

Thrombocytopenia is defined as an absolute platelet count <150.000/µL or a decrement greater than 50% from baseline value^[1]^. This is a common finding in critically ill patients classically associated with poor prognosis, prolonged hospital stay, higher risk and, ultimately, reduced survival^[2]^. Consequently, platelet count has been implicated as a prognostic marker for various severity scoring systems, such as Multiple Organ Dysfunction Score (MODS)^[3,4]^, Sequential Organ Failure Assessment (SOFA)^[5]^, Logistic Organ Dysfunction Score (LODS)^[6]^, and others^[7]^.

There are basically four mechanisms that can decrease platelet counts: decreased production, increased destruction (immune-or nonimmune-mediated), hemodilution and sequestration (as in hypersplenism)^[1,2,8,9]^. In the general intensive care unit (ICU) scenario, thrombocytopenia may occur in up to 20% admissions, 35% of surgical hospitalizations and 45% of trauma admissions^[1]^. Considering cardiac surgery patients, thrombocytopenia is an extremely frequent finding, varying from 35% to 65% of all cases^[10]^, and patients after percutaneous coronary interventions (PCI) are also prone to develop major thrombocytopenia, *i.e*. <100.000/µL, in 2.4-9.2% of cases^[11-13]^.

Causes for thrombocytopenia following cardiac surgery are believed to be multifactorial, with cardiopulmonary bypass (CPB) installation as the main culprit. However, with the development of off-pump coronary artery bypass grafting (CABG) and more recent transcatheter valve procedures, such as transcatheter aortic valve replacement (TAVR), new questions arose regarding thrombocytopenia in a post-cardiac surgery setting. Even without the use of CPB, platelet count continues to drop, which rendered further doubts not only as to the origin of this problem, but also to its clinical implications. To date, there are very few studies available which tackle this issue, with some showing strong correlation between the occurrence of severe thrombocytopenia after TAVR and higher mortality rates^[14]^.

When we consider the valve-in-valve (ViV) scenario and the incidence of thrombocytopenia, even less can be said. Although transcatheter ViV procedures is a well-established option for treatment of degenerated bioprosthesis in high and extreme-risk patients^[15]^, and the incidence of thrombocytopenia is high after these procedures, there are no available data in the literature addressing this problem. Therefore, little is known about this phenomenon and its correlation with unfavorable events.

## METHODS

We carried on a retrospective, single-centre, database analysis study of all consecutive patients who underwent transcatheter ViV implantation from May 2015 until March 2017. A total of 30 patients underwent transcatheter ViV procedures at our institution during this period, with the majority of them performed through transapical access (82%). One patient who had transcatheter mitral ViV and concomitant TAVR, and one patient who received two concomitant ViVs for mitral and tricuspid valve degeneration, were not included in this study, leaving the remainder of 28 patients. Of these, 5 were performed on degenerated bioprosthetic valves on aortic position, 18 on mitral and 5 on tricuspid. Four of the five patients in the tricuspid group had transjugular access, and one patient was accessed through a transatrial right anterior thoracotomy. All patients who underwent ViV procedures were deemed high or extreme risk for conventional approach, and were thoroughly discussed by our heart team. Surgical procedures were conducted in a hybrid operating room, using fluoroscopic and echocardiographic guidance. The transcatheter valve utilized in all patients was the Braile Inovare^®^ prosthesis, a balloon expandable valve platform with a chromium-cobalt stent frame, widely used in Brazilian experience^[16,17]^.

Pre- and postoperative data were collected and the platelet curves were elaborated, starting with the first value upon ICU admission, and taking into account daily values of platelet counts until the 10^th^ postoperative day. Postoperative bleeding was also quantified and correlated with the drop in platelet values, as well as the occurrence of other adverse events, such as paravalvular regurgitation, acute renal failure and death. All definitions were in accordance with the Valve Academic Research Consortium (VARC-2) standardized endpoint definitions for TAVR consensus document^[18]^.

Furthermore, data was collected from all conventional reoperations for degenerated bioprosthetic valve replacement, performed at our institution during the same period to serve as a control group. A total of 146 patients were analyzed and 74 were included, after patients with active infective endocarditis, combined procedures (*i.e*.: CABG + valve replacement), previous valve repair and non-replacement, use of mechanical valves at any point, septic shock, previous hematologic disease, pregnant women and those with perioperative mortality were excluded. Postoperative platelet curves between both groups were compared and correlated with clinical outcomes.

### Statistical Analysis

For the variables with homogeneous distribution, parametric tests were performed and the results were presented in mean and standard deviation. As for the variables with non-homogeneous distribution, we performed non-parametric tests and the results are presented in median and interquartile range. Chi-square test and Fisher's exact test were used to compare the categorical variables. Analysis of the quantitative variables was performed by comparing means using the t-test or Kruskal-Wallis test. We considered as statistically significant differences the results with values of *P*<0.05. Statistical analysis were conducted using IBM SPSS Statistics^®^ 20 for Windows (Chicago, IL, United States).

## RESULTS

Thrombocytopenia occurred in 100% of patients who underwent transcatheter ViV implantation, regardless of the position where the degenerated bioprosthesis was originally placed. Seven (25%) patients experienced mild thrombocytopenia, with the nadir of platelet counts between 100.000-150.000/µL, 15 (54%) showed moderate thrombocytopenia (50.000-100.000/µL) and 6 (21%) presented with severe thrombocytopenia postoperatively (<50.000/µL), as shown in Table 1. Within this group, there was no statistical difference between the degree of thrombocytopenia presented and the occurrence of adverse events, such as death, major bleeding, acute renal failure and sepsis. Regarding baseline characteristics, there was also no significant factor that could predict a higher risk of postoperative thrombocytopenia.

**Table 1:** Baseline and postoperative characteristics of valve-in-valve patients according to the degree of thrombocytopenia, in absolute values.

	General	Mild thrombocytopenia (≥100.000/µL) 7 patients	Moderate thrombocytopenia (<100.000/µL) 15 patients	Severe thrombocytopenia (≤50.000/µL) 6 patients	*P*-value
**Preoperative**					
Age	60 (16-82)	59 (16-80)	60 (34-81)	62 (21-78)	0.796
EuroSCORE II	7.45 (2,86-32.87)	9.85 (1.52-32.93)	8.75 (2.52-26.87)	10,10 (1.52-26.46)	0.943
NYHA I-II	9 (32.14)	3 (42.85)	4 (26.6)	2 (40)	0.753
NYHA III-IV	19 (67.85)	4 (57.14)	11 (73.3)	3 (60)	0.753
Chronic renal failure	15 (53.57)	4 (57.14)	8 (53.3)	3 (50)	0.967
Atrial fibrillation/flutter	17 (60.71)	5 (71.4)	10 (66.6)	2 (33.3)	0.3
CAD	10 (35.71)	3 (42.8)	4 (26.6)	3 (50)	0.543
DM	7 (25)	2 (28.6)	4 (26.6)	1 (16.6)	0.856
CABG	7 (25)	2 (28.6)	4 (26.6)	1 (16.6)	0.856
Previous prosthesis stenosis	12 (43)	2 (29)	4 (40)	4 (67)	0.361
Previous prosthesis insufficiency	16 (57)	5 (71)	9 (60)	2 (33)	0.361
No preoperative thrombocytopenia	16 (57.14)	5 (71.4)	8 (53.3)	3 (50)	0.663
Mild preoperative thrombocytopenia	8 (28.6)	2 (28.6)	5 (33.3)	1 (16.6)	0.732
Moderate preoperative thrombocytopenia	4 (14.28)	0 (0)	2 (13.3)	2 (33.3)	0.17
**Postoperative**					
Absent or mild paravalvular regurgitation	25 (92)	6 (85.7)	15 (100)	4 (80)	0.071
Moderate paravalvular regurgitation	2 (7.4)	1 (14.2)	0 (0)	1 (20)	0.071
Death	5 (17.8)	1 (14.28)	2 (13.33)	2 (33.3)	0.572
Major bleeding	2 (7.1)	1 (14.3)	0 (0)	1 (16.7)	0.196
Acute renal failure	17 (60.7)	3 (42.8)	10 (66.6)	4 (66.6)	0.542
Sepsis	11 (39.28)	2 (28.6)	7 (46.6)	2(33.3)	0.677

CABG=coronary artery bypass grafting; CAD=coronary artery disease; DM=diabetes mellitus; NYHA=New York Heart Association

### Platelet Curves for ViV Patients

**Fig. 1:**
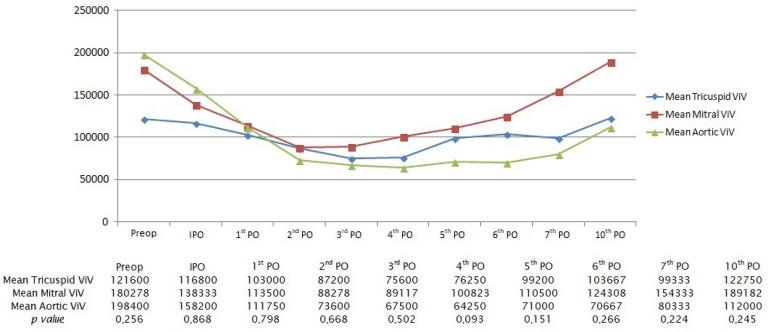
Mean platelet curves for all patients who underwent a valve-in-valve procedure. IPO=immediate postoperative period; PO=postoperative day; ViV=valve-in-valve

In every position where the transcatheter valve was implanted, patients presented with a drop in platelet counts already at the immediate postoperative period, as shown in Figure 1.

Of note, four out of five patients who had a ViV implanted in tricuspid position were preoperatively thrombocytopenic (average preoperative platelet count of 121.600/µL), two with mild and two with moderate thrombocytopenia. The nadir of the tricuspid curve occurred at the 3^rd^ postoperative day, and by the 10^th^ day, the mean platelet count had reached preoperative values, but remained below the normal threshold of 150.000/µL. In this subgroup of patients, the nadir of the platelet curve represented a 36.8% drop from baseline.

In the mitral ViV subgroup, the preoperative platelet count was normal, with an average of 180.278/µL. Thrombocytopenia was observed in the immediate postoperative period, with the nadir occurring at the 2^nd^ postoperative day. Platelet counts began to rise from the 3^rd^ day onward, reaching normal values at the 7^th^ postoperative day. In turn, the nadir of the curve represented a 45.3% fall in relation to preoperative values.

Aortic ViV patients also presented with normal preoperative platelet counts, and had the highest values amongst the three subgroups: 198.400/µL. These patients also experienced a platelet drop in the immediate postoperative period; however, thrombocytopenic values were only reached at the 1^st^ postoperative day and persisted for longer. The nadir of the curve occurred in the 4^th^ day and, until the 10^th^ day, regular preoperative levels had not yet been reached, unlike the other two subgroups. The aortic ViV subgroup of patients presented with the most expressive drop in platelet count: 62.3%.

Despite differences in absolute and percentage values between the three subgroups, they were not statistically significant.

### Platelet Curves for ViV *vs.* Conventional Reoperations

The control group consisted of 74 patients who underwent conventional reoperations to replace degenerated bioprosthesis at our institution, during the same period. Of these, 31 were submitted to redo aortic valve replacement and 43 to redo mitral valve replacement. No isolated tricuspid redo patients could be included because these were all congenital heart disease patients, who usually underwent combined procedures, or had other complex cardiac repairs previously done, which frequently utilized synthetic material and had to be excluded.

The comparison between patient's preoperative characteristics in each group is shown in Tables 2 and 3. As expected, ViV patients were older and had more comorbidities, such as chronic renal failure, coronary artery disease and previous CABG. These differences were statistically significant and became more evident in the mitral group comparison. Although EuroSCORE II was higher in both ViV subgroups, this aspect showed no statistical difference.

**Table 2:** Preoperative characteristics comparison between 18 mitral ViV patients and 43 conventional mitral reoperations.

	Mitral ViV	Mitral conventional reoperation	*P*-value
Age	63.9	54.7	0.025
EuroSCORE II	11.48%	7.59%	0.075
NYHA I-II	4 (22.2)	10 (23.2)	1
NYHA III-IV	14 (77.8)	33 (76.7)	1
Chronic renal failure	12 (66.7)	13 (30.23)	0.011
Atrial fibrillation/flutter	12 (66.7)	26 (60.5)	0.775
CAD	6 (33.3)	4 (9.3)	0.051
DM	5 (27.8)	4 (9.3)	0.108
Previous CABG	4 (22.2)	1 (2.3)	0.024
Previous prosthesis stenosis	6 (33.3)	29 (67.4)	0.022
Previous prosthesis insufficiency	12 (66.7)	31 (72.1)	0.547

CABG=coronary artery bypass grafting; CAD=coronary artery disease; DM=diabetes mellitus; NYHA=New York Heart Association

**Table 3:** Preoperative characteristics comparison between 5 aortic ViV patients and 31 conventional aortic reoperations.

	Aortic ViV	Aortic conventional reoperation	*P*-value
Age	79	61.8	<0.001
EuroSCORE II	8.78%	7.28%	0.161
NYHA I-II	2 (40)	13 (41.9)	1
NYHA III-IV	3 (60)	17 (54.8)	1
Chronic renal failure	3 (60)	9 (29)	0.3
Atrial fibrillation/flutter	2 (40)	10 (32.3)	1
CAD	4 (90)	9 (29)	0.047
DM	2 (40)	5 (16.1)	0.24
Previous CABG	3 (60)	4 (12.9)	0.04
Previous prosthesis stenosis	4 (90)	23 (74.2)	1
Previous prosthesis insufficiency	1 (20)	23 (74.2)	0.034

CABG=coronary artery bypass grafting; CAD=coronary artery disease; DM=diabetes mellitus; NYHA=New York Heart Association

**Fig. 2:**
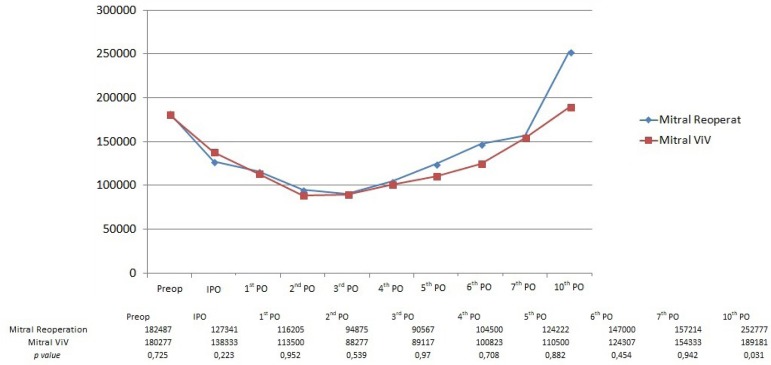
Comparison between mean platelet values for conventional mitral reoperation and mitral valve-in-valve (VIV) patients.

The comparison between conventional mitral valve reoperation and mitral ViV showed very similar platelet curves, as shown in Figure 2. Thrombocytopenia was present at the immediate postoperative period as well, the nadir occurred at day 3 and platelet levels returned to baseline by day 7. The drop in platelet count represented 47.73% from baseline, and the difference between both groups was not statistically significant until the 7^th^ postoperative day. However, by the 10^th^ day, conventional redo patients had a higher platelet counts than ViV, with statistical significance. Only one (2%) patient in the conventional surgery group did not experience postoperative thrombocytopenia.

Differences between conventional and transcatheter groups were more pronounced in aortic patients. Conventional surgery patients presented thrombocytopenia in the immediate postoperative period and the nadir was sooner, on the 3^rd^ postoperative day. The curves crossed and recovery of platelet count was faster in the conventional group, reaching normal preoperative levels by the 6^th^ day and presenting a considerable difference by the 10^th^ postoperative day.

The nadir of the curve was on the 4^th^ day and represented a drop of 52.4% in platelet count.

**Fig. 3:**
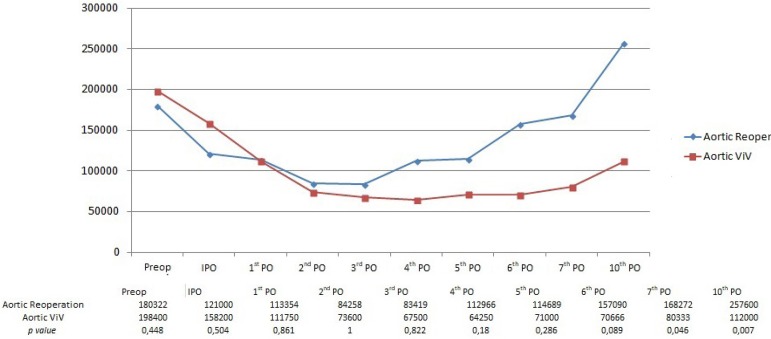
Comparison between mean platelet values for conventional aortic reoperation and aortic valve-in-valve (VIV) patients.

From the end of the fist week until the 10^th^ day, the highest level of thrombocytopenia in the ViV group was statistically significant, as shown in Figure 3. In the aortic control group, only 3 (7.5%) patients remained with normal platelet counts in the postoperative period.

### Predictors and Outcomes

To further refine the data analysis, we divided all ViV patients considering the degree of thrombocytopenia presented, according to each individual's initial platelet count (drop ≥50% or <50%), instead of accounting only for the absolute lowest platelet value. This is shown in Table 4, where 16 (57%) patients presented with a drop in platelets ≥50% and 12 (43%) had less than a 50% drop.

**Table 4:** Baseline and postoperative characteristics of valve-in-valve patients according to the degree of thrombocytopenia in relation to each individual's baseline platelet value and proportional drop in platelet count.

	Drop <50% (n=12)	Drop >50% (n=16)	*P*-value
**Preoperative**			
Age	54	65	0.205
EuroSCORE II	10 (2-33)	9 (3-26)	0.478
NYHA I-II	4 (33.3)	5 (31.25)	0.612
NYHA III-IV	8 (66.6)	11 (68.75)	0.612
Chronic renal failure	6 (50)	9 (56.25)	0.521
Atrial fibrillation/flutter	8 (66.6)	9 (56.25)	0.435
CAD	3 (25)	7 (43.75)	0.268
DM	1 (8.3)	6 (37,5)	0.091
CABG	3 (25)	4 (25)	0.666
Previous prosthesis stenosis	6 (50)	6 (37.5)	0.391
Previous prosthesis insufficiency	6 (50)	10 (62.5)	0.391
Absent or mild paravalvular leak	11 (91.6)	15 (93.75)	0.389
Moderate paravalvular leak	1 (8.3)	1 (6.25)	0.389
No preoperative thrombocytopenia	3 (25)	13 (81.25)	0.004
Mild preoperative thrombocytopenia	6 (50)	2 (12.5)	0.04
Moderate preoperative thrombocytopenia	3 (25)	1 (6.25)	0.196
**Postoperative**			
Absent or mild paravalvular regurgitation	11 (91.6)	15 (93.75)	0.389
Moderate paravalvular regurgitation	1 (8.3)	1 (6.25)	0.389
Death	2 (16.6)	3 (18.75)	0.643
Major bleeding	1 (8.3)	1 (6.250)	0.683
Acute renal failure	7 (58.3)	10 (62.5)	0.565
Sepsis	5 (41.6)	6 (37.5)	0.565

CABG=coronary artery bypass grafting; CAD=coronary artery disease; DM=diabetes mellitus; NYHA=New York Heart Association

There were also no statistically significant differences between patient's characteristics in both groups, except for the preoperative platelet count. A normal platelet count from baseline was significantly correlated with a greater fall in platelet levels, over 50%. Paradoxically, patients who already had mild to moderate preoperative thrombocytopenia experienced a less pronounced drop in platelet count. Nevertheless, a drop over 50% was not correlated with higher risk of mortality, major bleeding, acute renal failure, or other studied outcomes.

## DISCUSSION

In this retrospective study, we aimed to correlate pre- and postoperative characteristics of ViV patients with postoperative thrombocytopenia, which occurred in all of our patients. There were expected preoperative differences between patients in the ViV group and in the conventional reoperation group. Naturally, those who underwent ViV procedures had been deemed as high/extremely high risk for conventional surgery by our heart team, and therefore were older, with more comorbidities, a higher incidence of chronic renal failure and previous CABG. Nevertheless, the EuroSCORE II values, although higher for the ViV group, did not show a statistically significant difference between both groups.

Surprisingly, although thrombocytopenia is regarded as multifactorial, it has, for a long time, been attributed mainly to the installation of CPB, since upon initiation of bypass platelets drop to approximately 50% from baseline^[12]^. The unavoidable hemodilution that succeeds, the sheer stress to the platelets, by roller or centrifugal pumps, and extracorporeal plastic tubing interaction, have been commonly charged with causing their ultimate destruction^[12,19]^. Other well-established factors associated with thrombocytopenia in cardiac surgery are the use of intra-aortic balloon pumps (IABP), sepsis and drug-related, mainly heparin-induced thrombocytopenia (HIT), a rare phenomenon with incidence around 1%^[11-13]^.

However, thrombocytopenia occurred in a similar fashion in patients submitted to transcatheter ViV implants, which were not placed in CPB. Platelet curves for conventional surgery and mitral ViV implants were particularly similar, with no statistical difference until the 7^th^ postoperative day, whereas curves comparing procedures for the aortic position showed significant difference, with a more pronounced and prolonged platelet drop in ViV implants.

Therefore, we may infer that, although multifactorial, CPB may not necessarily be the main cause of thrombocytopenia after valvular heart surgery, and platelet interaction with the bioprosthetic valve components such as bovine pericardium, Teflon felts, chromium-cobalt or nitinol stent frames, may play a much more important role. This could explain why aortic ViV patients experienced a higher degree of thrombocytopenia in comparison to mitral ViV.

Physiologically, transvalvular pressure and velocity gradients are higher in aortic than in mitral position, which could justify higher stress levels to platelets and translate into greater platelet degradation and consumption.

To the authors' knowledge, this is the first study to try to correlate the degree of thrombocytopenia after ViV procedures and the incidence of adverse events. Jilaihawi et al.^[11]^ conducted a similar study regarding thrombocytopenia, comparing 246 TAVR patients (200 transfemoral and 46 transapical) with 57 high-risk surgical aortic valve replacement (SAVR) patients. They found very similar platelet curves between both groups, with a greater decrease in the SAVR group, unlike our ViV aortic group comparison. Major thrombocytopenia occurred in over one third of patients in the TAVR group, which proved to be benign and self-limited in most cases. As in our study, they were unable to find a significant relationship between moderate/severe thrombocytopenia and adverse events, when platelet count normalizes before the end of the first postoperative month. However, in the rare cases in which thrombocytopenia was found to be persistent (lasting longer than 30±10 days), this was an independent predictor of mortality (HR 3,27).

In a comparable fashion, Dvir et al.^[14]^ conducted a study with 488 TAVR patients seeking an association between acquired thrombocytopenia and clinical outcomes. Postoperative thrombocytopenia was extremely common and transient, with 90.2% of patients presenting with no or mild thrombocytopenia at discharge, but 36.1% showed significant thrombocytopenia.

Interestingly, they found a nadir of platelet count <50.000/µL to be a highly specific marker for 30-day mortality. The occurrence of severe thrombocytopenia, analyzed through multivariate logistic regression, proved to be and independent risk factor for one-year mortality (OR 3,44).

The authors were able to correlate the degree of thrombocytopenia with the following adverse events at 30 days: cardiovascular death, sepsis, major bleeding, greater units of blood transfusion, acute kidney injury (AKI) and prolonged ICU stay. Therefore, they propose that thrombocytopenia can be used as an excellent prognostic marker for adverse short- and long-term outcomes after TAVR.

In our study population, there was no significant correlation between the degree of thrombocytopenia and the incidence of adverse events, and no preoperative or immediate postoperative factors could predict the occurrence of thrombocytopenia. Jilaihawi et al.^[11]^ found significant association between moderate/severe persistent thrombocytopenia and hepatic cirrhosis, male sex and preoperative thrombocytopenia. Dvir et al.^[14]^, on the other hand, found the following characteristics associated with severe thrombocytopenia after TAVR: preoperative thrombocytopenia, leukopenia, vascular access complications, major bleeding, multiple transfusions, AKI, and sepsis. In contrast, our ViV group analysis showed that patients with normal preoperative platelet counts were at a higher risk of presenting with a greater drop in platelet counts >50% than patients who already presented with mild or even moderate thrombocytopenia.

Two other studies focused on thrombocytopenia after TAVR were conducted by Flaherty et al.^[20]^, who analyzed 90 TAVR patients who received the Edwards SAPIEN valve; and McCabe et al.^[21]^, which compared 112 TAVR patients with 105 balloon aortic valvuloplasties (BAV) patients. Flaherty et al.^[20]^ identified that thrombocytopenia occurred in 79% of post-TAVR patients, and independent preoperative predictors for moderate/severe thrombocytopenia were baseline thrombocytopenia, leaner body mass, smaller aortic valve area, higher peak aortic jet velocity and worse renal function. The occurrence of major thrombocytopenia was a predictor for major vascular complications and major bleeding. The study conducted by McCabe et al.^[21]^, on the other hand, found that, although there was a significant difference in the prevalence of thrombocytopenia in TAVR patients (69%) in comparison to BAV (37%), this did not translate into any major clinical outcomes, except for an increased use of hemocomponents. The possibility of severe or persistent thrombocytopenia following TAVR remains a major concern, since bleeding following TAVR is independently associated with increased mortality.

Furthermore, there may be more on the issue of thrombocytopenia than meets the eye. A Polish study, conducted by Mitrosz et al.^[22]^, analyzed 32 TAVR patients and found that the only factor which positively correlated with drop platelets was the amount of iodinated contrast used during the procedure. This aspect was not taken into consideration in any of the other studies mentioned above.

Interestingly, in all TAVR studies, there was a considerable number of patients who did not present thrombocytopenia after the procedure. However, in our ViV study, all patients developed postoperative thrombocytopenia. These were all high-risk patients, with similar baseline characteristics, whose differences can be credited mainly to the surgical procedure itself (ViV rather than TAVR) and to the type of valve prosthesis utilized (Braile INOVARE, Medtronic COREVALVE and Edwards SAPIEN). Considering differences in surgical bioprosthetic valves as an example, Piccardo et al.^[23]^ have already shown a higher incidence of thrombocytopenia after surgical implant of the Freedom Solo valve in comparison to Carpentier-Edwards Perimount (25% *vs.* 3%, respectively), which did not translate into deleterious events.

## CONCLUSION

Thrombocytopenia after ViV procedures remains an extremely frequent finding, with unknown origins. In our study population, this phenomenon occurred in all patients; however, the degree of thrombocytopenia could not be associated with the incidence of adverse outcomes, probably due to a limited number of patients. Thrombocytopenia, therefore, did not prove itself to be a marker of worse prognosis in our ViV population.

Since previous studies, with larger series of patients, have shown positive implications of the decrease in platelet counts and higher postoperative risk in TAVR patients, we believe that further investigation of this matter in the ViV cohort should be encouraged.

**Table:** 

Authors' roles & responsibilities	
RCS	Substantial contributions to the conception or design of the work; or the acquisition, analysis, or interpretation of data for the work; drafting the work or revising it critically for important intel-lectual content; final approval of the version to be published
LP	Substantial contributions to the conception or design of the work; or the acquisition, analysis, or interpretation of data for the work; drafting the work or revising it critically for important intellec-tual content; final approval of the version to be published
GV	Substantial contributions to the conception or design of the work; or the acquisition, analysis, or interpretation of data for the work; drafting the work or revising it critically for important intellec-tual content; final approval of the version to be published
JA	Agreement to be accountable for all aspects of the work in ensuring that questions related to the accuracy or integrity of any part of the work are appropriately investigated and resolved; final ap-proval of the version to be published
LLA	Substantial contributions to the conception or design of the work; or the acquisition, analysis, or interpretation of data for the work; final approval of the version to be published
HR	Substantial contributions to the conception or design of the work; or the acquisition, analysis, or interpretation of data for the work; final approval of the version to be published
ROS	Substantial contributions to the conception or design of the work; or the acquisition, analysis, or interpretation of data for the work; final approval of the version to be published
FT	Drafting the work or revising it critically for important intellectual content; final approval of the version to be published
PMAP	Drafting the work or revising it critically for important intellectual content; final approval of the version to be published
JHP	Drafting the work or revising it critically for important intellectual content; final approval of the version to be published
FBJ	Final approval of the version to be published
